# Development and validation of a novel disulfidptosis-related gene signature for prediction of survival and immune microenvironment in osteosarcoma by WGCNA analysis

**DOI:** 10.1007/s12672-025-04146-y

**Published:** 2025-12-04

**Authors:** Yibin Zheng, Hang Cai, Hongbin Huang, Guolin Wu, Anlong Ren, Jincan Su, Jianhang Bao, Fengqing Wu

**Affiliations:** Department of Orthopedics, Yiwu Central Hospital, 699 Jiangdong Road, Yiwu, 322000 Zhejiang China

**Keywords:** Osteosarcoma, Disulfidptosis, Prognosis, Risk model, BTN3A1

## Abstract

Disulfidptosis was reported to be associated with the malignant progression of various tumors. This study was aimed to investigate the prognostic significance of disulfidptosis-related genes (DRGs) in osteosarcoma (OS). Ten previously reported core disulfidptosis genes were used for consensus clustering and WGCNA analyses. A total of 338 disulfidptosis-related genes (DRGs) were identified. Then, uni-COX, LASSO and multi-COX analyses were conducted, identifying 5 prognosis-related DRGs (BTN3A1, CEBPA, KCNAB2, TBX21, and MYC). A prognostic DRGs risk signature based on the five genes were constructed and validated. OS patients were divided into high and low risk groups by risk scores. K-M plots and t-ROC curves showed that patients with high-risk scores had worse prognosis. Patients in the high-risk group had lower abundance of immune checkpoint-related genes, including CD274 (PD-L1), LAG3, PDCD1LG2 (PD-L2), and BTLA. Besides, patients in the high-risk group exhibited lower IC50 values for vorinostat, elesclomol, OSI-906, pyrimethamine, thapsigargin, and doxorubicin, but a higher IC50 value for cisplatin, compared to those in the low-risk group, indicating differential drug sensitivities. Additionally, analysis of immune checkpoint blockade (ICB) response revealed that patients in the high-risk group had a lower predicted response rate to immunotherapy. The mRNA expression levels of 4 DRGs including BTN3A1, KCNAB2, TBX21 and CEBPA in OS cells were significantly lower than those in hFOB1.19 cells. Subsequent experiments revealed that BTN3A1 protein was expressed at low levels in OS cells. Furthermore, overexpression of BTN3A1 significantly suppressed OS cell proliferation, migration, and invasion. In summary, we established a robust DRGs signature comprising BTN3A1, CEBPA, KCNAB2, TBX21, and MYC, which showed strong prognostic value and predictive potential for immune status and drug sensitivity in OS. Notably, functional experiments confirmed that BTN3A1 acted as a tumor suppressor in OS, highlighting it as a promising therapeutic target.

**Supplementary Information:**

The online version contains supplementary material available at 10.1007/s12672-025-04146-y.

## Introduction

Osteosarcoma (OS) was a malignant and aggressive bone tumor, with high rate of mortality and disability [1]. Children and adolescents were the most susceptible population to OS. Tumor resection combined with adjuvant chemotherapy represents the standard treatment for patients with osteosarcoma (OS), from which many primary OS patients have received significant clinical benefits [2]. Unfortunately, the patients with distant metastasis, such as lung metastasis, remains a poor prognosis despite the diligent efforts in treatment[3]. The lack of the understanding of genetic complexity of OS and missing regarding the prognosis-related mechanisms of OS may led to an inappropriate treatment [4]. And accurately assessing the prognosis of OS patients is difficult attributed of its heterogeneity [5]. Therefore, it is urgent to find new biomarkers for OS patient stratification and improve the accuracy of individualized treatment.

Disulfidptosis was a modality of program cell death (PCD) reported by Xiaoguang Liu et al.[6] And this type of cell death was mainly triggered by disulfide stress due to the aberrant accumulation of intracellular disulfides [7]. In recent years, disulfide metabolism had been considered to be closely related with the development of cancers. Reactive oxygen species (ROS) and lipid peroxides accumulate when tumor cells are exposed to hypoxic and nutrient-poor environments [8]. Protective molecules, such as glutathione, are activated to mitigate the detrimental effects. Studies have confirmed that tumors synthesized a large amount of glutathione by up-regulating the expression of SLC7A11 and then promoting the transport of cystine into cells [9]. Recent studies have demonstrated that SLC7A11 is markedly upregulated in OS cells and tissues, where it enhances glutathione (GSH) synthesis and protects tumor cells against intracellular oxidative stress [10]. Functional evidence indicates that targeting SLC7A11 can inhibit the proliferation and invasion of OS cells by triggering ferroptosis, highlighting its critical role in tumor progression [11]. Intriguingly, elevated SLC7A11 expression has been reported to increase tumor cell vulnerability to disulfide stress, suggesting that activating disulfidptosis may represent a novel therapeutic strategy to overcome resistance and metastasis in OS [6]. However, the potential relationship between disulfidptosis and OS remains largely unexplored, underscoring the need for further investigation.

A Strong relationship may exist between disulfide ptosis and tumor development. And some bioinformatic studies have shown that disulfidptosis-related genes (DRGs) are related to tumor prognosis, immune infiltration and drug resistance at present [12–14]. Therefore, identifying the relationship between DRGs and OS was helpful for finding new therapeutic target.

In this study, we identified 338 DRGs by the weighted correlation network analysis (WGCNA) and then established a novel disulfidptosis-related gene (DRGs) prognostic signature for survival prediction and guiding clinical treatment of OS patients. Subsequently, the accuracy of the model in prognosis prediction was evaluated, and the function enrichment analysis, immune infiltration, drug sensitivity analysis and immune checkpoint analysis were performed. Finally, we found that BTN3A1 was downregulated in OS cells. Furthermore, overexpression of BTN3A1 significantly suppressed the proliferative, migratory, and invasive capacities of OS cells. It also provides a new perspective on the individualized therapy of OS.

## Materials and methods

### Raw data acquisition

**Fig. 1:**
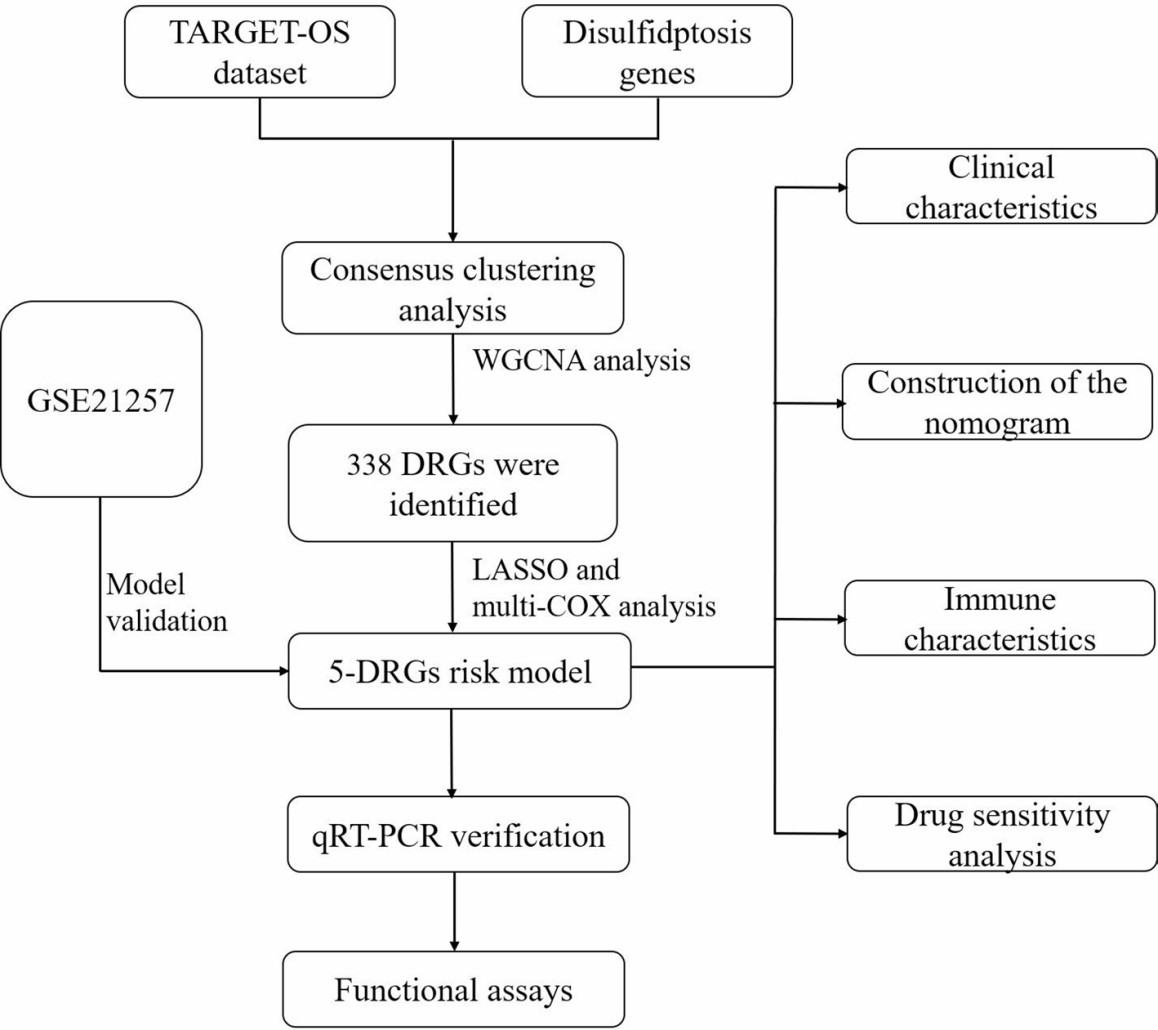
The flow chart of our study

The RNA-seq and clinical data of 85 OS patients from TARGET database that with complete information were obtained from the Xena website (https://xenabrowser.net/). And the format of RNA-seq data was transformed from log_2_(FPKM + 1) into log_2_(TPM + 1). Besides, data of 57 OS patients in GSE21257 dataset were downloaded from the Gene Expression Omnibus (GEO) database (https://www.ncbi.nlm.nih.gov/geo/). In addition, ten key disulfidptosis genes, including SLC7A11, GYS1, LRPPRC, NCKAP1, NDUFA11, NDUFS1, NUBPL, OXSM, RPN1 and SLC3A2, were obtained from the study of Boyi Gan et al.[6]. The workflow was shown in Fig. 1.

### Consensus clustering and survival analysis

Firstly, the prognostic predictive powers of those ten disulfidptosis genes were assessed by univariate COX (Uni-COX) analysis. And the consensus unsupervised clustering analysis based on those genes were performed by using the “ConsensusClusterPlus” package. Patients were divided into different groups according the optimal *k* values. K-M plot was drafted to evaluate the survival status of different groups.

### Identification of disulfidptosis-related genes using WGCNA analysis

**Fig. 2:**
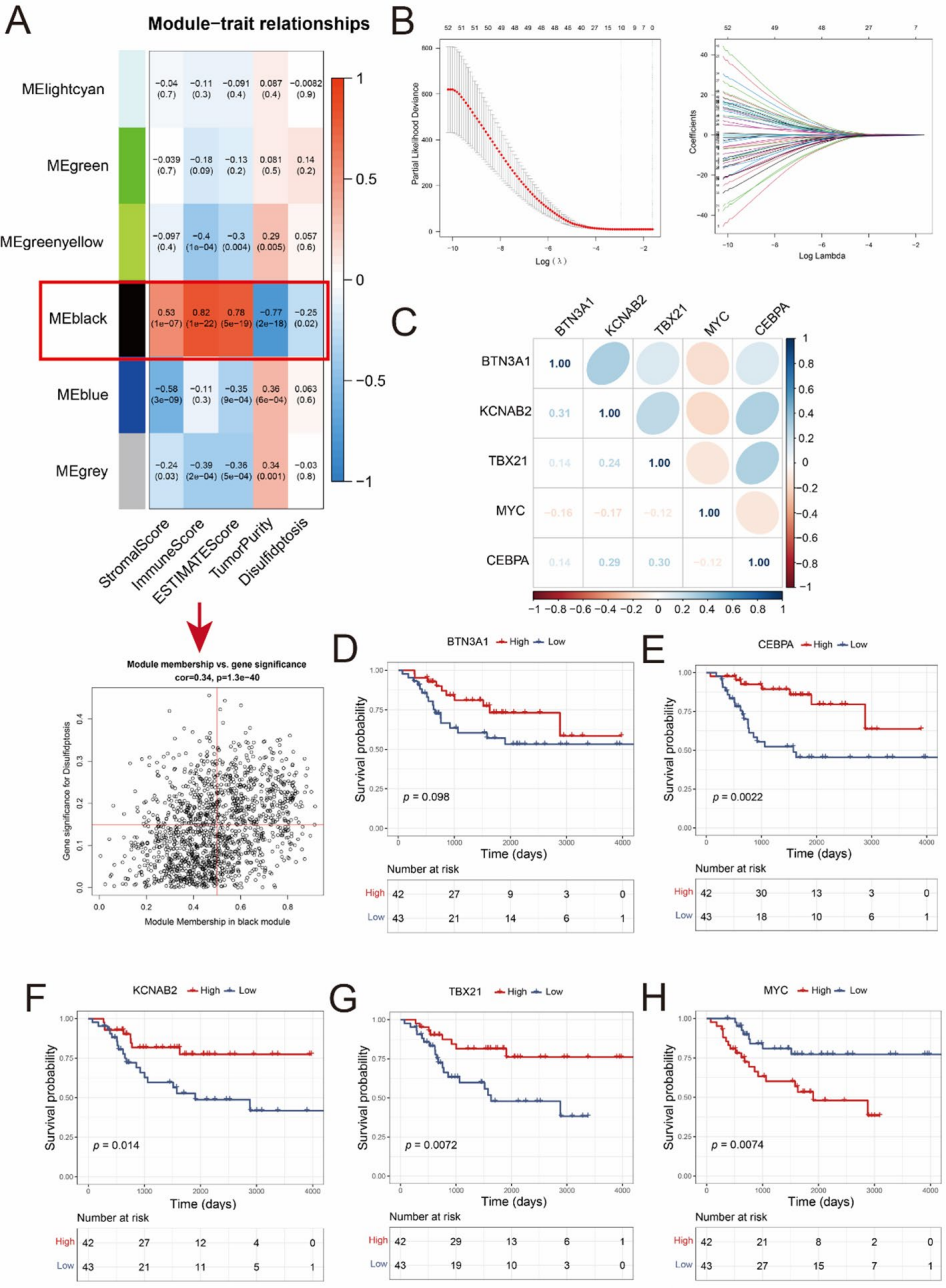
Identification of DRGs signature in the TARGET cohort of OS. **A** Identification of DRGs by WGCNA analysis. **B** LASSO and COX analysis of DRGs. **C** Correlation between the expression levels of these 5 genes (Pearson correlation analysis). **D–H** Overall survival of patients between different groups layered according to expression of prognostic DRGs (Log-rank test)

We carried out the single sample Gene Set Enrichment Analysis (ssGSEA) analysis to calculate the disulfidptosis scores of each patient based on the expression level of the ten disulfidptosis genes. The enrichment statistic (ES) value of ssGSEA score in patient were calculated by the “GSVA” package. Subsequently, WGCNA analysis was performed to find out the DRGs according to disulfidptosis scores [15]. In short, the outliner samples were detected (Sup Fig. 1A). And clustering dendrogram of samples based on the Euclidean distance was performed (Sup Fig. 1B). The Pearson correlation analysis was applied to construct the weighted adjacency matrix of mRNA. The soft-thresholding parameter was set at 9 (Sup Fig. 1C). And we constructed the topological overlap matrix (TOM) according to the generated adjacency matrix. All mRNA related genes were then classified based on TOM dissimilarity. Finally, the relationship between the disulfidptosis scores and tumor/immune scores were analyzed. According the result of WGCNA analysis, genes in MEblack module with correlation >0.5 and gene significance correlation >0.15 was regarded as DRGs (Fig. 2A).

### Construction and validation of DRGs risk model

The prognosis-related DRGs were then found out by uni-COX analysis *via* the “survival” package. Then, LASSO analysis and multi-COX were performed to narrow the number of the candidate genes and to obtain the regression coefficient (β value). Pearson correlation analysis was used to clarify the correlation of genes by the “corrplot” package. And we constructed the risk score model as follows:

$${\text{DRG scores }} = \sum \beta _{{\text{i}}} \times {\text{G}}_{{\text{i}}}$$

(β was the regression coefficient and G represented the different gene.)

According to the DRG scores, patients in TARGET dataset and GEO dataset were divided into two groups: high- and low-DRG score groups. The K-M plot and the tROC curve were performed by R software to determine the predictive power of risk model.

### Clinical utility of signature and construction of nomogram model

The patients of TARGET-OS were divided into two subgroups based the clinical features: age (< 18 or ≥ 18 years old), gender (male or female) and metastatic status (M0 or M1). And the K-M plots were used to evaluate the predictive power of the DRGs risk model in different subgroups. Moreover, the independent predictive ability of the DRGs risk model was assessed by uni- and multi-Cox regression analyses. Then, a novel nomogram was constructed to improve the model performances. The calibration plots were drawn to display the deviation between the predictive and actual risk values by “rms” R package. ROC curves were drafted to evaluate the predictive power of nomogram model by the “survivalROC” package. And the decision curve analysis (DCA) curves were conducted by “stdca” package [16].

### Gene set enrichment analysis

The “limma” package was performed to find out different expressed genes (DEGs) between those two DRGs risk groups [17]. Then, we performed the gene set enrichment analysis (GSEA), including GSEA-KEGG, GSEA-GO and GSEA-Pathway, to infer the common functions of these genes [18].

### Tumor microenvironment analysis

The tumor microenvironment (TME) significantly affects tumor cell proliferation and malignant metastasis [19]. Therefore, the “estimate” R package was used for assessing the immune scores. Then, the ssGSEA was performed to analysis the proportion of immune cells between two DRGs risk groups [20].

### Therapeutic sensitivity analysis, immune checkpoint and immune respond analysis

The “pRRophetic” package was utilized to calculate the half-maximal inhibitory concentrations (IC50) values of various chemotherapy drugs and targeted agents. Additionally, we assessed the differences of expression of immune checkpoint genes between two groups through student’s t-test and visualized the results using “ggplot2” package. Besides, the Tumor Immune Dysfunction and Exclusion (TIDE) scores and immune checkpoint blockade (ICB) responses in different patients were analyzed by using TIDE (http://tide.dfci.harvard.edu/login/).

### Cell culture

The hFOB1.19, MG-63, 143B, Saos-2 and U2-OS cells were obtained from the Cell Bank, Chinese Academy of Sciences (Shanghai, China). The hFOB1.19 cells were incubated at 33.5 °C, while the OS cells at 37 °C. And cells were cultured in DMEM-high glucose medium, MEM medium and McCOY’S 5 A medium with 10% fetal bovine serum (FBS), respectively.

### Lentiviral package and trasduction

Lentiviral vector for BTN3A1 overexpression (pLV3-CMV-BTN3A1(human)-3×FLAG-CopGFP-Puro) was purchased from MiaoLing Plasmid (China). The plasmids were first transformed into Escherichia coli DH5α for amplification, and purified using standard plasmid extraction protocols. For viral production, the lentiviral transfer plasmid was co-transfected with the packaging plasmids psPAX2 and pMD2.G into HEK293T cells using established transfection methods [21]. Viral supernatants were collected 48 h post-transfection, filtered to remove cell debris, and concentrated by centrifugation. Aliquots of the concentrated virus were stored at − 80 °C for subsequent experiments. For transduction, 143B and HOS cells were seeded in 6-well plates and exposed to the viral supernatant overnight. The medium was replaced 48 h after infection, and fluorescence microscopy was used to evaluate transduction efficiency. Stable cell lines were generated through puromycin selection, expanded, and cryopreserved. A lentiviral construct encoding luciferase (GeneCare, China) was prepared following the same workflow.

### Quantificational real time-PCR (qRT-PCR)

Total RNA of cells was isolated using Total RNA Extraction Reagent (EZBioscience, the USA) and then reversed to cDNA by HiScript Reverse Transcriptase (Vazyme, China). qRT-PCR analysis was performed as previously study [22]. 18 s was used as the internal reference gene. The primers used were shown in Table 1.

**Table 1:** The primers of different genes

Genes		Sequence
TBX21	F	5ʹ- CGCCAGGAAGTTTCATTTGG -3ʹ
	R	5ʹ - TCACCTCAACGATATGCAGC -3ʹ
CEBPA	F	5ʹ- CCACGCCTGTCCTTAGAAAG -3ʹ
	R	5ʹ- CCCTCCACCTTCATGTAGAAC -3ʹ
BTN3A1	F	5ʹ- TCCGAATACACAACGTCACAG -3ʹ
	R	5ʹ- TGCCTCTCATGATCACAGATG -3ʹ
KCNAB2	F	5ʹ- CAAGGCTGAAGTGGTACTGG -3ʹ
	R	5ʹ- TCGATTATGTGCTTCCTGGAC -3ʹ
18s	F R	5ʹ- GCAATTATTCCCCATGAACG -3ʹ 5ʹ- GGGACTTAATCAACGCAAGC -3ʹ

### Western blot

The cell pellet was collected, total protein was extracted using strong RIPA lysis buffer (Beyotime, China), and protein concentration was determined using BCA. Equal amounts of protein were adding into SDS-PAGE and separated. Then, the proteins were transferred onto methanol-activated PVDF membranes. After blocking with 5% skim milk for 1 h at room temperature, the PVDF membranes were washed with PBST and incubated with primary antibody at 4 ℃ overnight. Then, the membranes were washed with PBST for 3 times and incubated with secondary antibody for 1 h. Finally, the bands were recorded by BIO-RAD chemiluminescence kit. The primary antibody used as follows: anti-BTN3A1 (25221-1-AP, Proteintech, China) and anti-GAPDH (60004-1-Ig, Proteintech, China).

### CCK8 and colony formation assays

The cell proliferations were detected by CCK8 assay and colony formation assays as previous study [23].

### Edu assay

The cells were seeded in a 6-well plate and cultured for 24 h. The prepared EdU working solution was added to the cells and cultured for another 2 h. Subsequently, Edu staining was performed according to the operating instructions. Finally, the EdU staining image was obtained using a fluorescent inverted microscope and counted using ImageJ.

### Migration and invasion assays

The migration and invasion assays were performed as previous study [21].

### Statistical analysis

All statistical results were showed as the mean ± standard deviation (SD). The difference of two different groups was compared by student’s t-test analysis *via* GraphPad Prism 9.4.0 software. *P* < 0.05 was regarded as statistically significant difference.

## Results

### Consensus clustering and survival analysis

Firstly, we used the uni-COX analysis to identify whether these ten disulfidptosis genes (SLC7A11, GYS1, LRPPRC, NCKAP1, NDUFA11, NDUFS1, NUBPL, OXSM, RPN1 and SLC3A2) were associated with the OS prognosis. Regrettably, the results revealed that none of the them displayed a significant correlation with prognosis (Sup Table 1). Furthermore, consensus clustering analysis based on the ten genes were performed. The optimal *k* value of the analysis was determined to be 3 (Sup Fig. 1A). OS patients from TARGET dataset were then divided into three different clusters (Sup Fig. 1B). However, survival status between three clusters showed no significant differences (*p* = 0.91, Sup Fig. 1C).

### Identification of disulfidptosis-related genes signature in the TARGET cohort of OS

Due to the limited ability of cluster analysis in accurately distinguishing patients with varying prognosis, we identified prognostic-related DRGs for constructing risk model. Firstly, the ssGSEA analysis were performed to calculate the disulfidptosis scores and immune scores of patients from TARGET dataset. Then, the WGCNA analysis was performed and showed that the MEblack module was significantly related with the disulfidptosis and tumor immune microenvironment of OS (Fig. 2A). And a total of 338 genes were then identified as DRGs (Sup list 1). Subsequently, the uni-COX analysis was performed and identified 56 prognosis-related DRGs (Sup list 2). To minimize the impact of co-expression of genes, LASSO and COX analyses were carried out (Fig. 2B). Finally, we identified 5 prognosis-related DRGs (BTN3A1, CEBPA, KCNAB2, TBX21 and MYC). The pearson analysis demonstrated no significant correlation of the expression levels between these 5 genes (Fig. 2C). Furthermore, the K-M plots showed that those 5 genes had excellent independent prognostic predictive power when the median expression value was set as the cutoff value (Fig. 2D–I).

### Construction and validation of DRGs risk model

**Fig. 3:**
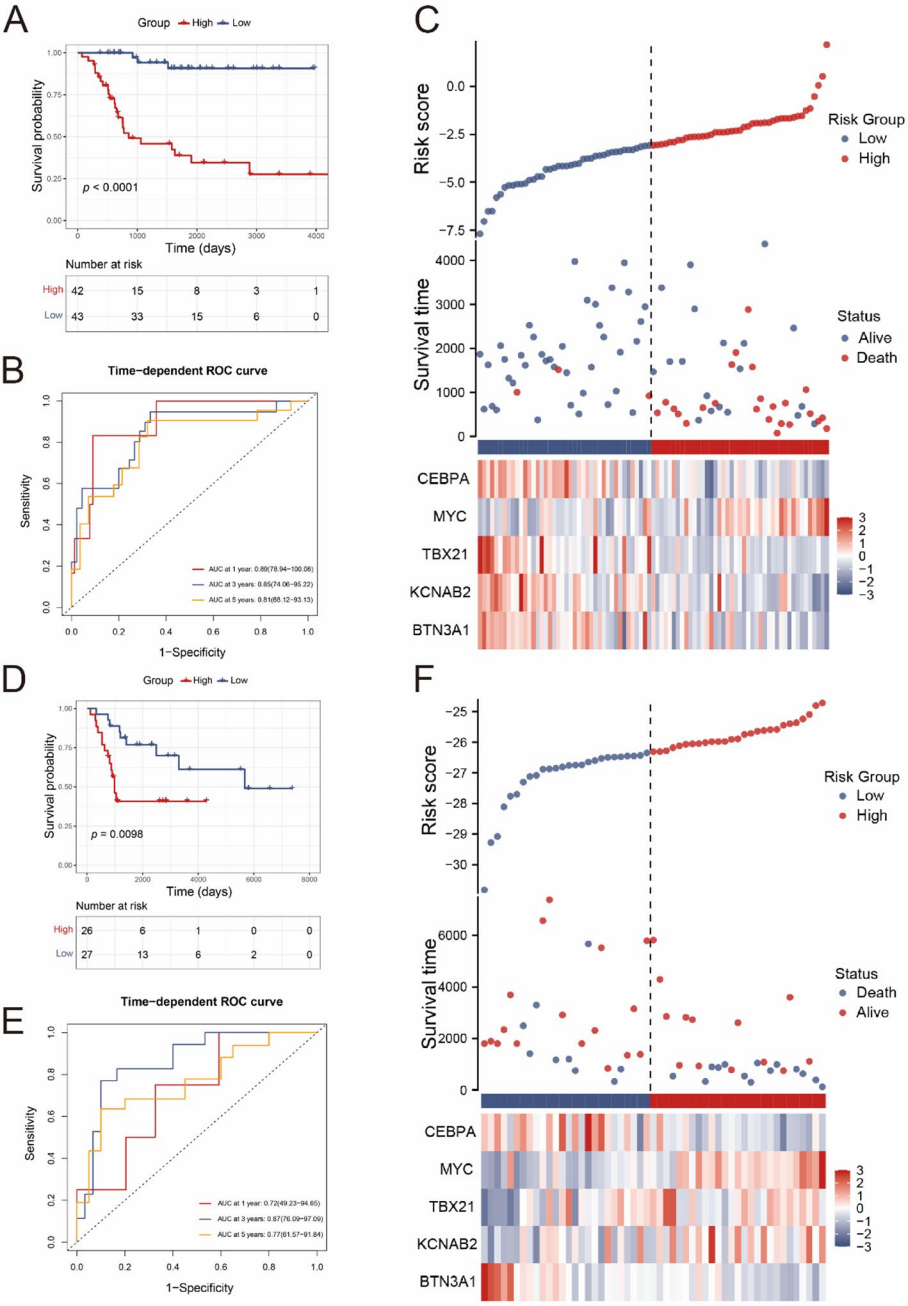
Construction and validation of DRGs risk model. **A** Survival curve of the OS patients of two groups in the training set (Log-rank test). **B** tROC of predictive performance of the model in the training set. **C** The layout of increase risk scores (up), clinical outcomes (middle) of patients, and heatmap (down) of these five genes of two groups in the training set. **D** Survival curve of two groups in the test set. **E** tROC of predictive performance of the model in the test set (Log-rank test). **F** The layout of increase risk scores (up), clinical outcomes (middle) of patients, and heatmap (down) of these five genes of two groups in and test set

According to the above results, we calculated the coefficients of genes by multi-COX analysis and then constructed the DRGs risk model: DRGs risk score = (−0.6691 × BTN3A1) + (−0.6750 × KCNAB2) + (−0.7154 × TBX21) + (0.6052 × MYC) + (−0.4686 × CEBPA). We calculated risk scores for patients in the TARGET dataset and stratified them into high- and low-risk groups based on the median score as the cutoff. The K-M survival analysis indicated that the survival status of patients in the high-risk score group was worse than in the low-risk score group (*p* < 0.0001, Fig. 3A). The tROC (time-dependent ROC) curve was then plotted to evaluate the accuracy of risk model in predicting 1-, 3- and 5-year overall survival, and the area under curve (AUC) values were 0.89, 0.85 and 0.81, respectively (Fig. 3B). And the relationship between the risk scores and survival time, survival status, and risk ranking were exhibited (Fig. 3C). A heatmap was plotted to display the expression patterns of the five prognostic DRGs (Fig. 3C). Furthermore, the GEO dataset, GSE21257, was obtained and then was used as an external dataset to assess the robustness of the model. We recalculated coefficients of 5 genes in testing set due to data format differences of gene expression between TARGET and GEO datasets. And patients from GEO dataset were divided into two groups. Similarly, the outcomes of patients in high-risk group were worse in the testing set (*p* = 0.0098, Fig. 3D). And the AUC of the DRGs risk model in GEO dataset was 0.72, 0.87 and 0.77 in predicting 1-, 3- and 5-year overall survival (Fig. 3E). Besides, there was strongly correlation between the risk scores and survival status and risk ranking, and gene expression patterns were significantly different between different risk groups (Fig. 3F). Taken together, the DRGs risk model exhibited excellent predictive performance on the test set as well as the training set.

### Analysis of clinical stratification and independent prognostic ability, and construction of nomogram

**Fig. 4:**
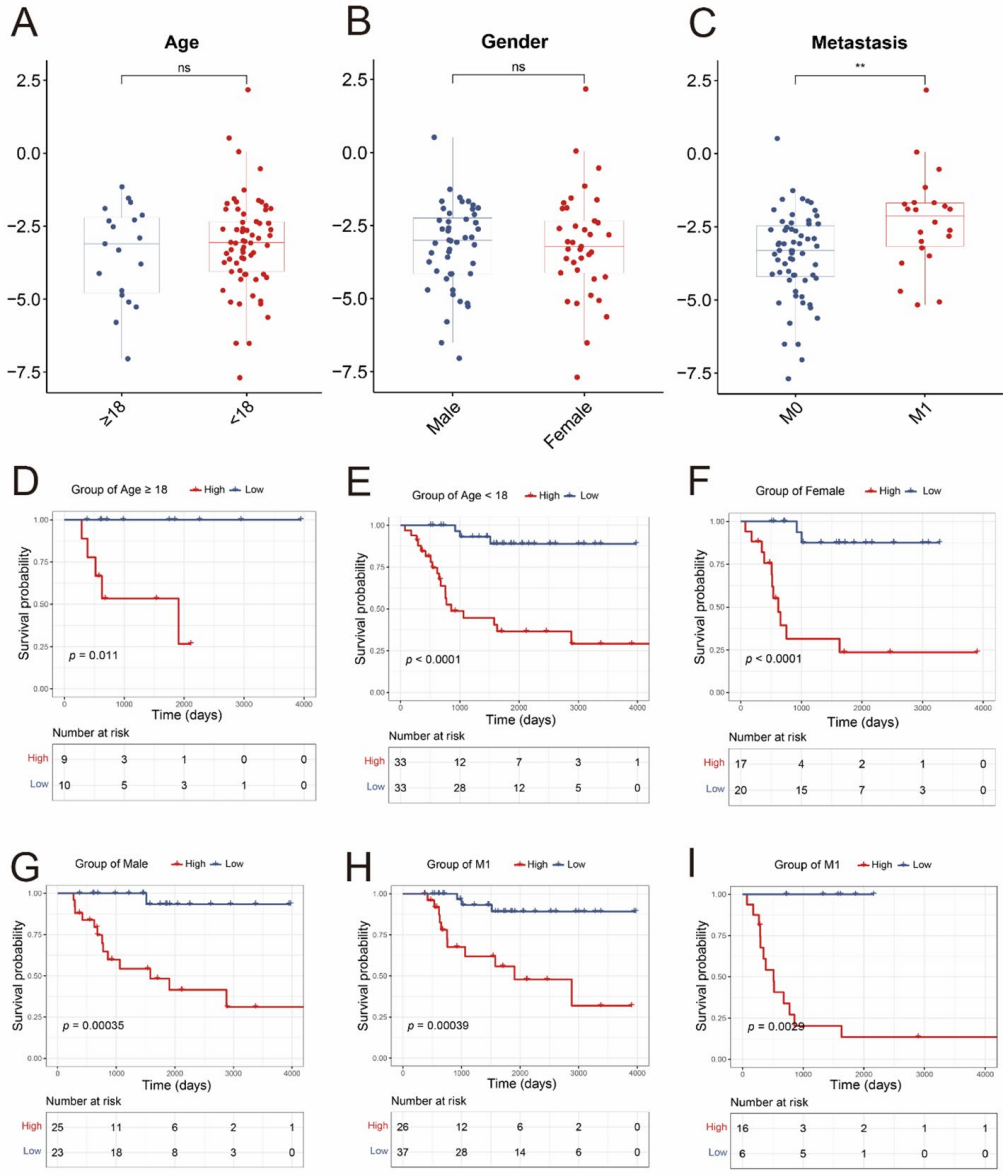
Clinical stratified analysis. **A–C** The DRGs risk scores between different clinical subgroups (student *t* test). **D–I** Survival curves of patients in different clinical subtypes (Log-rank test). ns, *P* > 0.05; **, *p* < 0.01

To further evaluate the predictive performance of the risk model, we stratified patients from TARGET dataset into different subgroups based on the gender (male or female), age (< 18 or ≥ 18 years old), and metastases status (M0 or M1), respectively (Fig. 4A–C). The risk scores in patients of different ages and genders showed no significant difference (Fig. 4A–B). And patients with metastases had higher risk scores (Fig. 4C). Besides, patients in high DRGs-score group had worse outcomes in all subgroups (Fig. 4D–I). These findings revealed the robust predictive ability of DRGs risk model across diverse populations.

**Fig. 5:**
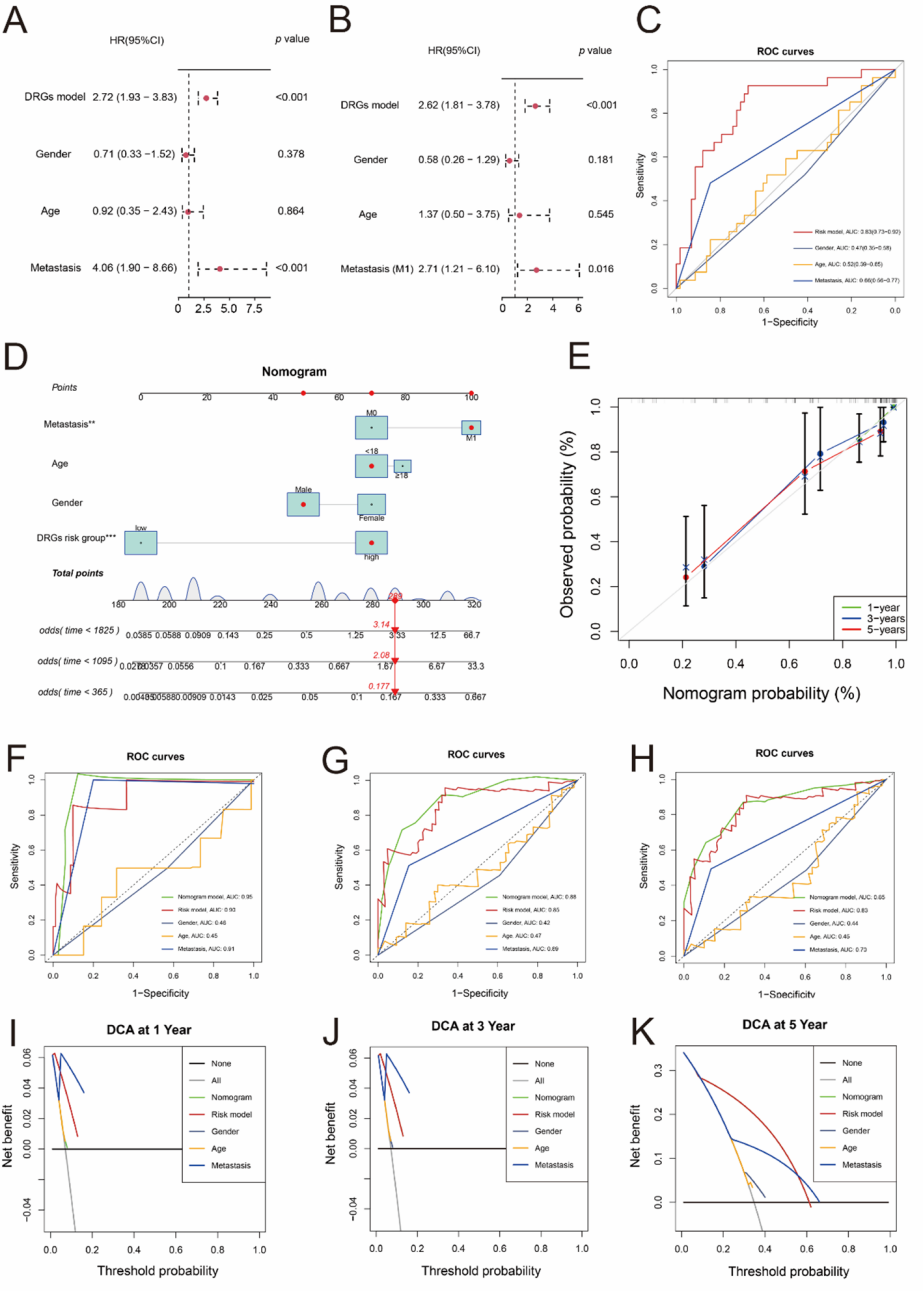
Independent prognostic analysis and nomogram construction. **A–B** The univariate (**A**) and multivariate Cox regression (**B**) to estimate the independence of the DRGs risk model. **C** ROC curves of DRGs risk model and clinical factors. **D** Nomogram model based on risk model and clinical features. **E** The calibration curve of nomogram model. **F–H** ROC curves of nomogram model, DRGs risk model and clinical factors at 1- (**F**), 3- (**G**) and 5-year (**H**). **I-K** DCA curves of nomogram model, DRGs risk model and clinical factors at 1- (**I**), 3- (**J**) and 5-year (**K**)

Furthermore, we performed the uni- and multi-Cox regression analyses to determine whether the DRGs risk model was as an independent prognosis predictor for OS. The result of uni-COX analysis showed that the DRGs risk score (HR = 2.72, *p* < 0.001) and metastasis status (HR = 4.06, *p* < 0.001) were closely related with the poor prognosis of OS patients (Fig. 5A). And multi-COX analysis demonstrated that the DRGs risk model (HR = 2.62, *p* < 0.001) was an independent prognosis indicator (Fig. 5B). The ROC curves showed that the DRGs risk model exhibited the best predictive power over other clinical factors (Fig. 5C).

Then, a novel nomogram model was established for improving the predictive power of DRGs risk model (Fig. 5D). The patients’ risk scores could be calculated by the nomogram model, and there was a good accordance between the survival probabilities predicted by the nomogram model and the actual survival probabilities at 1-, 3-, and 5-year (Fig. 5E). Besides, the AUC values of the nomogram model were 0.95, 0.88 and 0.85 at 1-, 3- and 5-year, which were higher than the AUC values of DRGs risk model and other clinical features (Fig. 5F–H). And the DCA curves showed that the nomogram model was better than other predictive factors (Fig. 5I–K).

### Analysis of immune microenvironment and immune checkpoint

**Fig. 6:**
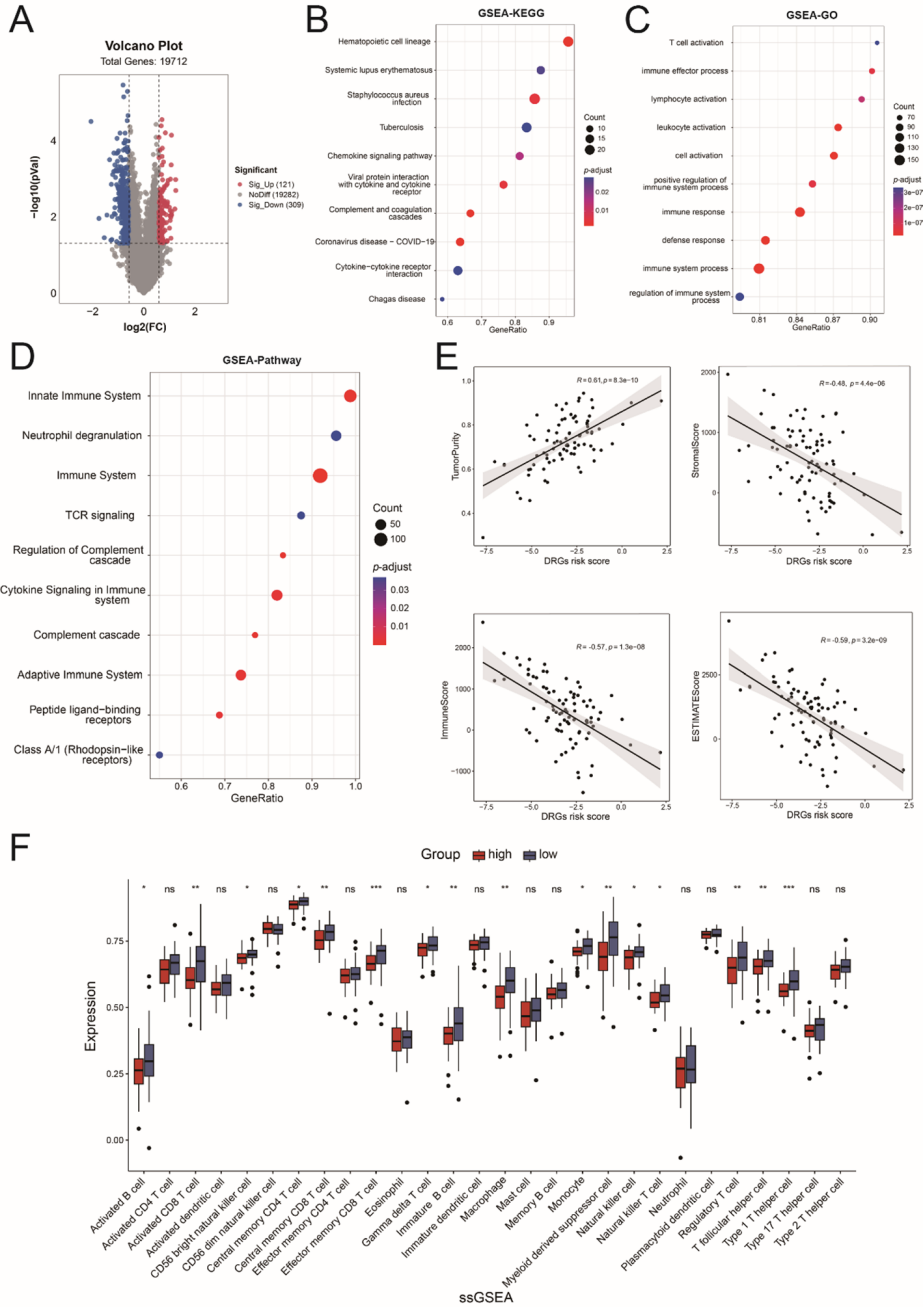
Function enrichment and analysis of immune microenvironment. **A** Volcano plot of DEGs between high and low risk groups. **B–D** GSEA-KEGG analysis (**B**) GSEA-GO biological process (**C**) analysis and GSEA-Pathway analysis (**D**) of theses DEGs. **E** The ESTIMATE analysis of OS patients from TARGET dataset. **F** Analysis of immune cell infiltration between the two groups (Wilcox test). ns, *P* > 0.05; *, *p* < 0.05; **, *p* < 0.01; ***, *p* < 0.001

To gain deeper insights into the functional distinctions among various risk groups, we conducted a differential genetic analysis between the high- and low-risk groups. 430 different expressed genes (DEGs) were identified, with 121 genes over-expressed and 309 genes down-regulated (Fig. 6A). The GSEA-KEGG analysis showed the function of “chemokine signaling pathway” and “cytokine-cytokine receptor interaction” were enriched (Fig. 6B). And the GSEA-GO analysis showed that the top ten biological processes were “T cell activation”, “immune effector process”, “lymphocyte activation”, “leukocyte activation”, “cell activation”, “positive regulation of immune system process”, “immune response”, “defense response”, “immune system process” and “regulation of immune system process” (Fig. 6C). Besides, the GSEA-Pathway analysis showed that the function of immune system was significantly different, with the pathway such as “innate immune system”, “neutrophil degranulation”, “immune system” and “TCR signaling” enriched (Fig. 6D). Therefore, we assessed the relationship between the DRGs risk scores and tumor immune microenvironments. As the DRGs risk scores increased, there was a notable correlation with the TumorPurity score (*R* = 0.61, *p* = 8.3e-10). In contrast, the StromalScore (*R* = −0.48, *p* = 4.4e-06), ImmuneScore (*R* = −0.57, *p* = 1.3e-08), and ESTIMATEScore (*R* = −0.59, *p* = 3.2e-09) exhibited significant decreases (Figs. 2A and 6E). In addition, the immune cells with tumor-killing effects, such as “activated CD8 T cell”, “CD56 bright natural killer cell” and “effector memory CD8 T cell” were sharply reduced in the high-risk group (Fig. 6F and Sup Fig. 3 A). Furthermore, the analysis of immune checkpoint revealed a lower abundance of immune checkpoint-related genes, including CD274 (PD-L1), LAG3, PDCD1LG2 (PD-L2), BTLA, CD200R1, CD40LG, CD48, HAVCR2 (TIM-3), LGALS9, and TNFSF14, in the high-risk group (Sup Fig. 3B). These results suggested that the DRGs-high group were more susceptible to the development of an immunosuppressive microenvironment. Collectively, the immune system function played a critical role in driving the OS malignant progression associated with cell disulfidptosis.

### Analyses of drug sensitivity and immune respond

**Fig. 7:**
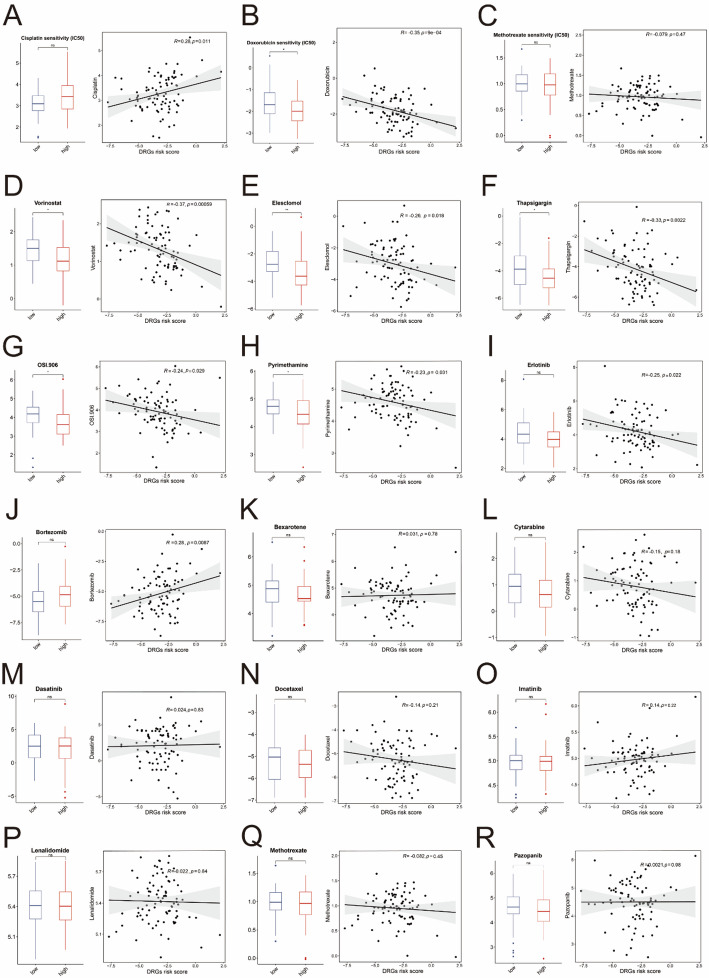
Analysis of Drug Sensitivity. **A–R** The predictive IC50 values of different chemotherapy drugs between two risk groups (student t test and *Pearson* correlation analysis). ns, *P* > 0.05; *, *p* < 0.05

Subsequently, we assessed the correlation between the DRGs risk scores and drug sensitivities as well as immune checkpoint abundances. The IC50 of cisplatin showed a gradual increase with rising DRGs risk scores, although the difference between the high-risk and low-risk groups was not statistically significant (Fig. 1A). Notably, the IC50 of doxorubicin exhibited a negative correlation with the Risk Score. And a significant difference was observed between the high- and low-risk groups (Fig. 1B). Besides, the OS patients in the high-risk group exhibited lower IC50 values for vorinostat, elesclomol, OSI.906, pyrimethamine and thapsigargin (Fig. 7D–H). And the decrease in IC50 values of these drugs was associated with increases in the DRGs risk scores (Fig. 7D–H). In addition, although the differences in IC50 values of erlotinib and bortezomib between the two groups were not statistically significant, the scatter plots revealed a clear trend of increasing or decreasing IC50 values corresponding to the risk scores (Fig. 7I–J). And there was no significant difference of the IC50 values of other drugs between two risk groups (Fig. 7C and K–R). Meanwhile, we analyzed TIDE scores and predicted ICB responses across for all patients. Interestingly, patients in the high-risk group exhibited lower TIDE scores (Sup Fig. 3C). However, analysis of immune checkpoint response rates revealed that the high-risk group had a lower likelihood of responding to ICB treatment (Sup Fig. 3D). These findings might provide insights for guiding clinical decision-making and personalized immunotherapy strategies.

### BTN3A1 inhibited the malignant phenotype of OS cells

**Fig. 8:**
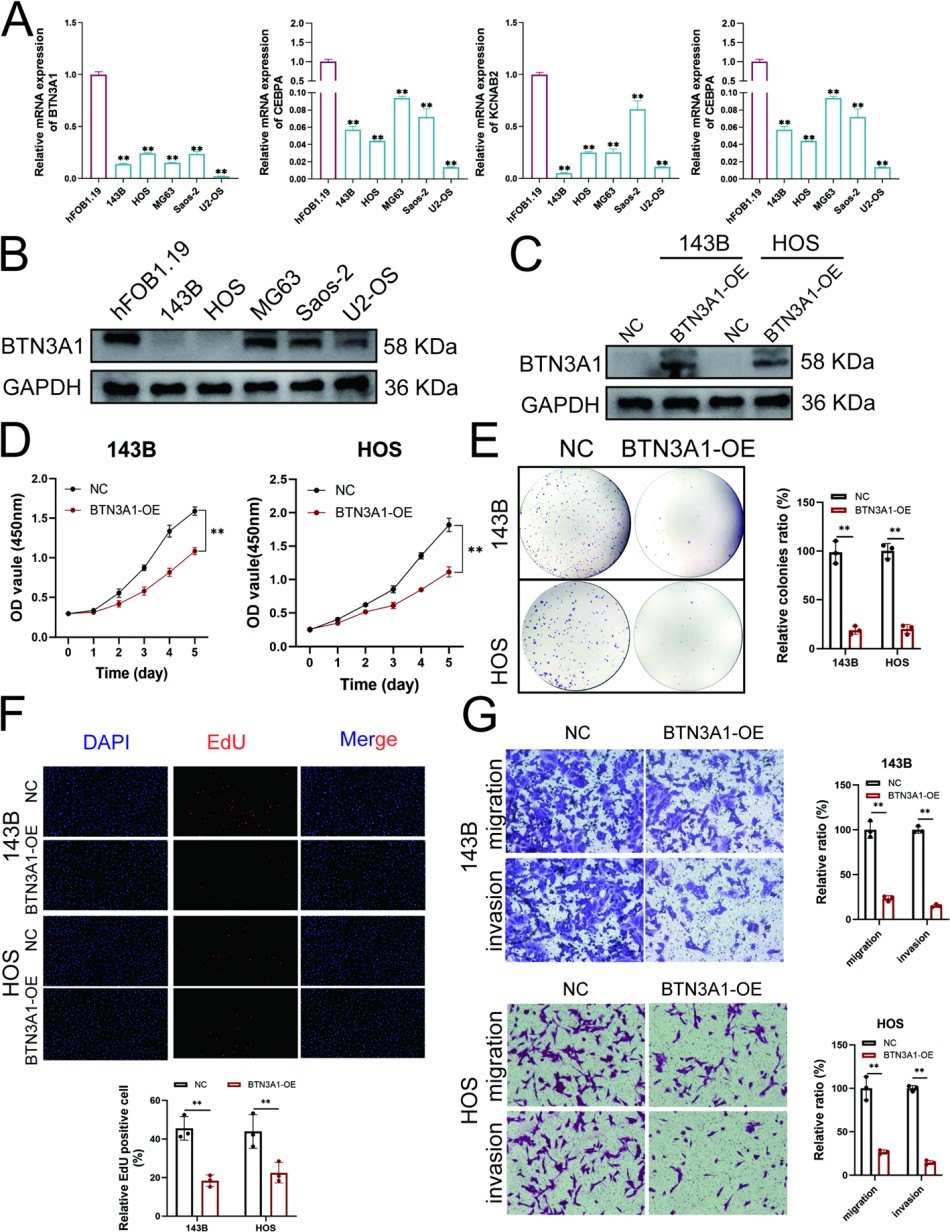
BTN3A1 inhibited the malignant phenotype of OS cells. **A** Relative mRNA expression levels of genes between Hfob1.19 and OS cells. **B** The BTN3A1 protein levels in hFOB1.19 and OS cells. **C** WB experiments confirmed that BTN3A1 was overexpressed in HOS and 143B cells. **D** CCK8 assay for detecting proliferation of OS cells overexpressing BTN3A1. **E** The representative results of cell clone colony formation assay. **F** The Edu assay results of BTN3A1 overexpressed cells. **G** The results of migration and invasion assays. **, *P* < 0.01 (student *t* test)

Among these five genes, MYC has been reported to be highly expressed in OS and is closely related to the malignant tendency of OS [24]. Therefore, we further determined the expression levels of other four genes in OS cells and hFOB1.19 cells. The results showed that the BTN3A1, KCNAB2, TBX21 and CEBPA, were down-expressed in the OS cells (Fig. 8A). Previous studies had demonstrated that CEBPA could inhibit the malignant progression of OS, whereas the roles of BTN3A1, KCNAB2, and TBX21 in OS remained unclear [25]. We further investigated the prognostic relevance of these three genes by the GSE21257 dataset. The results revealed that low expression of BTN3A1 was significantly associated with poor prognosis in OS patients, consistent with findings from the TARGET-OS dataset (Sup Fig. 4). Furthermore, BTN3A1 protein levels were markedly lower in OS cell lines compared to hFOB1.19 cells (Fig. 8B). To further explore its functional role, we generated HOS and 143B cell lines with stable overexpression of BTN3A1 using lentivirus (Fig. 8C). The CCK8 experiments showed that overexpression of BTN3A1 could significantly inhibit the proliferation of HOS and 143B cells (Fig. 8D). And the same results were obtained in the Edu and plate clone colony formation experiments (Fig. 8E-F). Transwell experiments showed that overexpression of BTN3A1 could significantly inhibit the migration and invasion of OS cells (Fig. 8G). These results suggested that BTN3A1 may be involved in inhibiting the malignant progression of osteosarcoma cells.

## Discussion

OS is an extremely aggressive and highly malignant tumor, characterized by a dismal prognosis [26]. Accurate prognosis prediction for patients is of utmost importance, as it enables the implementation of stratified treatment approaches tailored to individual case. Previous studies have demonstrated that DRGs are strongly associated with the prognosis of different tumor types, whereas few prognosis models have been conducted in OS [12, 13, 14, 27] Chen et al. performed cluster analysis on OS patients based on the disulfidptosis genes and found that two clusters of patients showed different prognosis [28]. However, our results showed that the patients were be divided into three subtypes according to the cluster analysis, and the prognostic characteristics showed no significant difference. To overcome this limitation, we applied WGCNA based on ten disulfidptosis genes and identified 338 DRGs, from which prognosis-related DRGs were further selected using LASSO regression and multivariate Cox analysis. A novel five-gene risk model was subsequently constructed, which proved to be a robust and independent predictor of OS patient outcomes. Importantly, survival analysis demonstrated that our model achieved superior predictive accuracy compared with the previously reported DRG-based model [28]. Beyond bioinformatic prediction, we further validated the expression and functional roles of selected gene in OS cells, confirming its biological relevance. This experimental verification not only strengthened the reliability of our model but also underscored its potential clinical translatability, suggesting that the five-gene signature might serve as a practical tool for risk stratification and therapeutic decision-making in OS.

The risk model was consisted of five prognostic related DRGs, including MYC, BTN3A1, KCNAB2, TBX21 and CEBPA. MYC is over-expressed in many human cancers and MYC-regulated transcription is highly dysregulated [29]. Previous studies have shown that MYC drives the proliferation and metastasis of OS, and inhibition of MYC can lead to OS suppression [30]. Furthermore, our study suggested that MYC might be closely related to disulfidptosis in OS. MYC has been reported to promote the metabolism reprograming of OS by enhancing the transcription of some important energy metabolism-related genes, especially regulating the glycolysis-related genes [31, 32]. The activation of cellular glucose metabolism by the MYC oncoprotein has been shown to enhance the production of NADPH, which may serve as a reducing agent to facilitate the resolution of disulfide bonds, thus suppressing disulfidptosis in cancer cells characterized by high levels of SLC7A11[33]. Besides, studies had found that CEBPA, as a transcription factor, could inhibit the oncogenic effect of the Runx3-Myc axis in OS cells [25]. However, the role of the other four genes in osteosarcoma remains poorly understood. As a member of the Kvβ superfamily, KCNAB2 has been reported to inhibit the proliferation and metastasis of lung cancer [34]. However, its function in other malignancies has yet to be fully elucidated. TBX21, a member of the T-box transcription factor family, is involved in modulating the biological behavior of several malignancies such as lung and colorectal cancers, and has been shown to be closely linked with tumor immune regulation [35]. BTN3A1, also referred to as CD277, is structurally related to the B7 family of costimulatory molecules. Emerging evidence indicates that members of the BTN3A family serve as critical regulators of Vγ9Vδ2 T cell function and are intricately involved in tumor immune modulation [36]. BTN3A1 has been reported to exhibit dysregulated expression in a wide range of malignancies. In this study, we found that BTN3A1 was down-expressed in OS cells. And the overexpression of BTN3A1 impaired the abilities the proliferation and invasion of OS cells, suggesting that it might serve as a potential therapeutic target in OS.

In our study, the GSEA analysis turned out that most of the DEGs between two risk groups were enriched in immune related pathway, which indicated that the five DRGs might be involved in regulating the OS immune microenvironment [37]. MYC, TBX21 and BTN3A1 were found to be associated with the immune infiltration in the tumor tissues, especially related to the infiltration of T cells [36–38].As is well-known, a high infiltration of immune cells into tumors indicates better patient survival rates [39]. Our risk score model was strongly correlated with tumor immune status. That patients in high-risk group had lower immune scores and higher tumor purity scores. In addition, the number of immune cells with antitumor ability dropped sharply in the high-risk group, specifically CD8^+^ and CD4^+^ T cells, which can recognize and kill tumor cells and can also produce various cytokines and chemokines to guide other immune cells to attack tumors [40, 41]. The reduction of CD8^+^ T cells might explain the poor immune status in high-risk group. Furthermore, the abundance of immune checkpoint genes was significant different between two risk groups. Immunosuppression-associated immune checkpoints were expressed at low levels in high-risk groups. These findings suggested that patients in the high-risk group might be poor responsive to immune checkpoint inhibitors [42]. We then examined the drug susceptibility of different risk groups. The result showed that the half maximal inhibitory concentration (IC50) of the most chemotherapeutic drugs, such as vorinostat, elesclomol, were lower in high-risk score group. In summary, this model might provide a valuable strategy for chemotherapy and immunotherapy with greater precision, particularly for patients in high-risk group. However, it should be noted that these predictions are derived from in silico analyses and may not directly reflect clinical responses.

This study has some limitations. First, we only detected the expression differences of these DRGs in the OS and hFOB1.19 cells. The expression levels of these five genes between normal and tumor samples should be evaluated. Second, biological functions of these genes in OS remain to be further studied.

## Conclusion

This study developed a clinicopathologically independent prognostic risk model comprising five DRGs, which could effectively predict the prognosis and response to immunotherapy in human OS. Besides, BTN3A1 was found as a tumor suppressor in OS, highlighting it as a promising therapeutic target. This research might deepen our understanding between disulfidptosis and OS.

## Supplementary Information

Supplementary Material 1.

## Data Availability

Availability of data and materialsAll data analyzed in this work came from public databases. The detailed analysis process can be obtained from the corresponding author on reasonable request.
