# Supplementary material for: Development and validation of a novel disulfidptosis-related gene signature for prediction of survival and immune microenvironment in osteosarcoma by WGCNA analysis
Source: Discov Oncol. 2025 Dec 4;17:47. doi: 10.1007/s12672-025-04146-y (PMC12779885; doi:10.1007/s12672-025-04146-y)
Supplement: Supplementary file 1 — Supplementary Material 1. [file 12672_2025_4146_MOESM1_ESM.pdf]

Supplementary Materials:

Sup Table 1 The uni-Cox Results of Ten Disulfidptosis Genes

Gene	p.value	HR (95% CI for HR)
GYS1	0.59	0.85 (0.47-1.5)
LRPPRC	0.17	1.6 (0.82-3)
NCKAP1	0.29	1.3 (0.77-2.3)
NDUFA11	0.85	1.1 (0.61-1.8)
NDUFS1	0.35	1.4 (0.71-2.6)
NUBPL	0.78	0.91 (0.48-1.7)
OXSM	0.36	1.4 (0.66-3.1)
RPN1	0.86	1.1 (0.53-2.1)
SLC3A2	0.76	0.9 (0.48-1.7)
SLC7A11	0.8	1 (0.73-1.5)

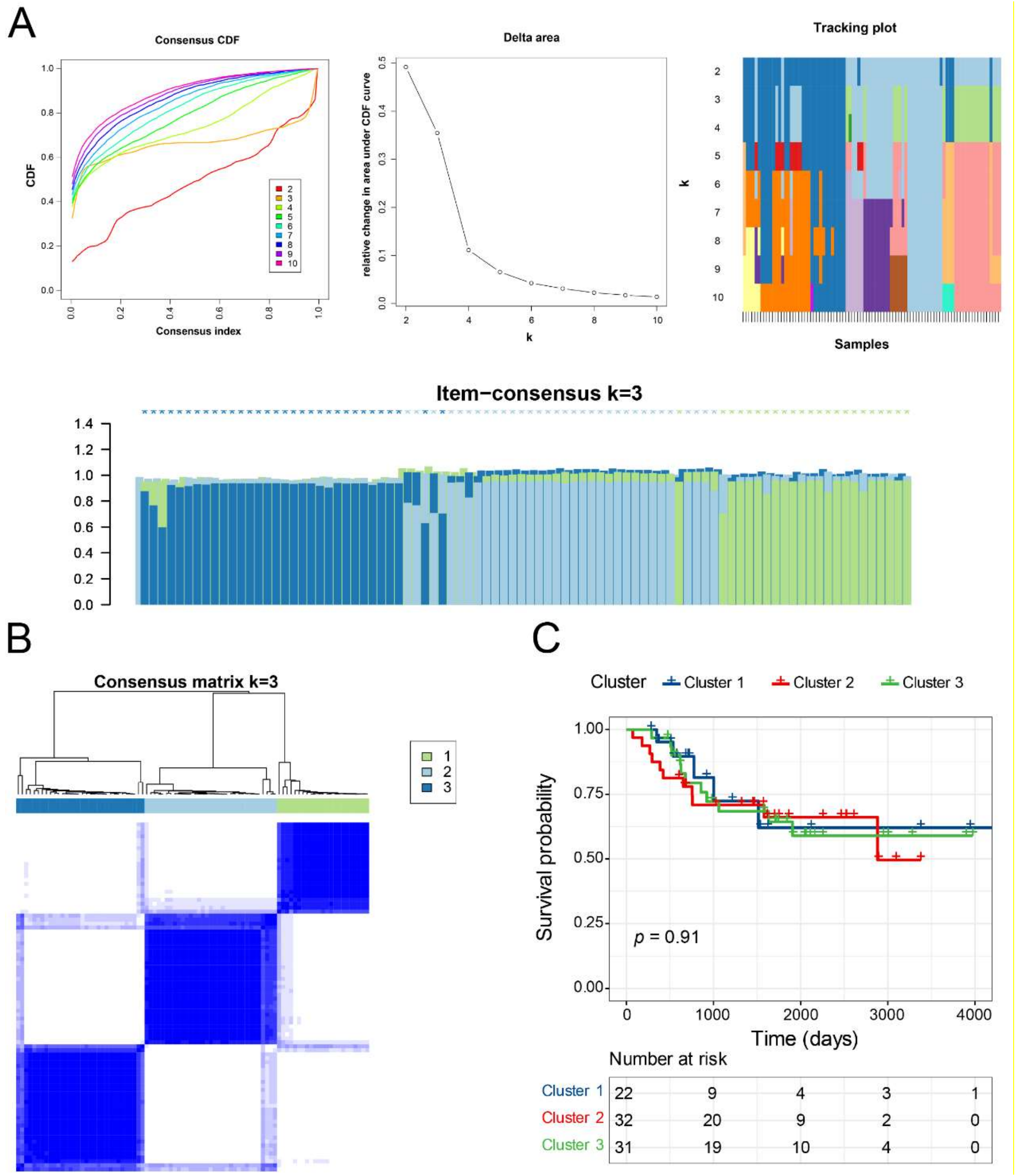

Sup Fig.1 Consensus clustering analysis based on ten disulfidptosis genes. (A) Optimal *k* value identified by "ConsensusClusterPlus" package. **(B)** Consensus matrix of patients in TARGET-OS when *k* = 3. **(C)** Survival curves of three different clusters.

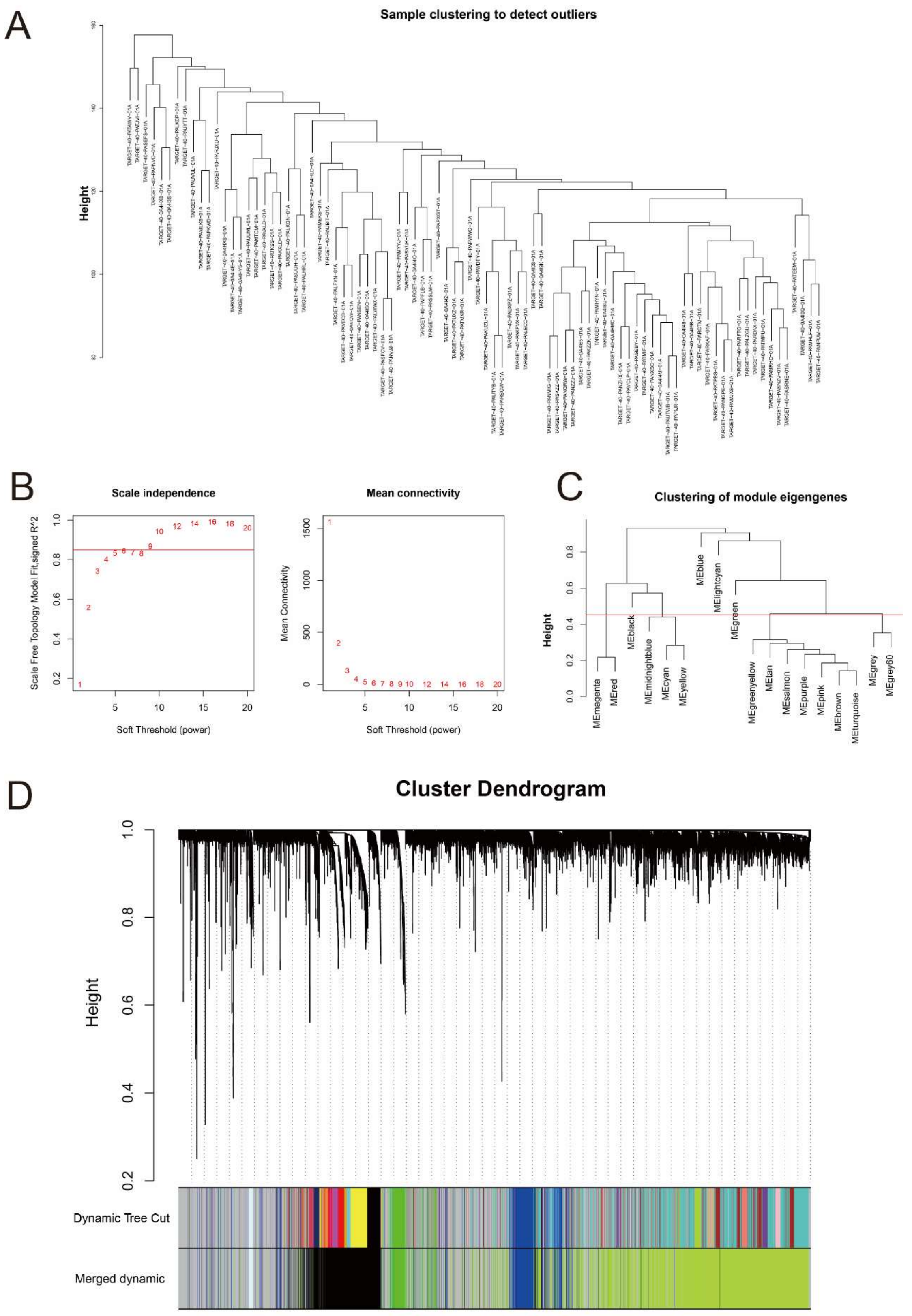

Sup Fig.2 The process of WGCNA analysis. (A) The sample clustering tree based on the WGCNA analysis of 85 OS tumors from the TARGET-OS cohort, and the hierarchical clustering by average link shows no outliner samples. **(B)** Soft-thresholding value was designated as 9 based on the scale-free fit index (left panel,

Scale-free $R^2 = 0.85$) and mean connectivity (right panel). **(C)** Clustering of module eigengenes when the height value = 0.45. **(D)** Hierarchical clustering dendrogram of module identification.

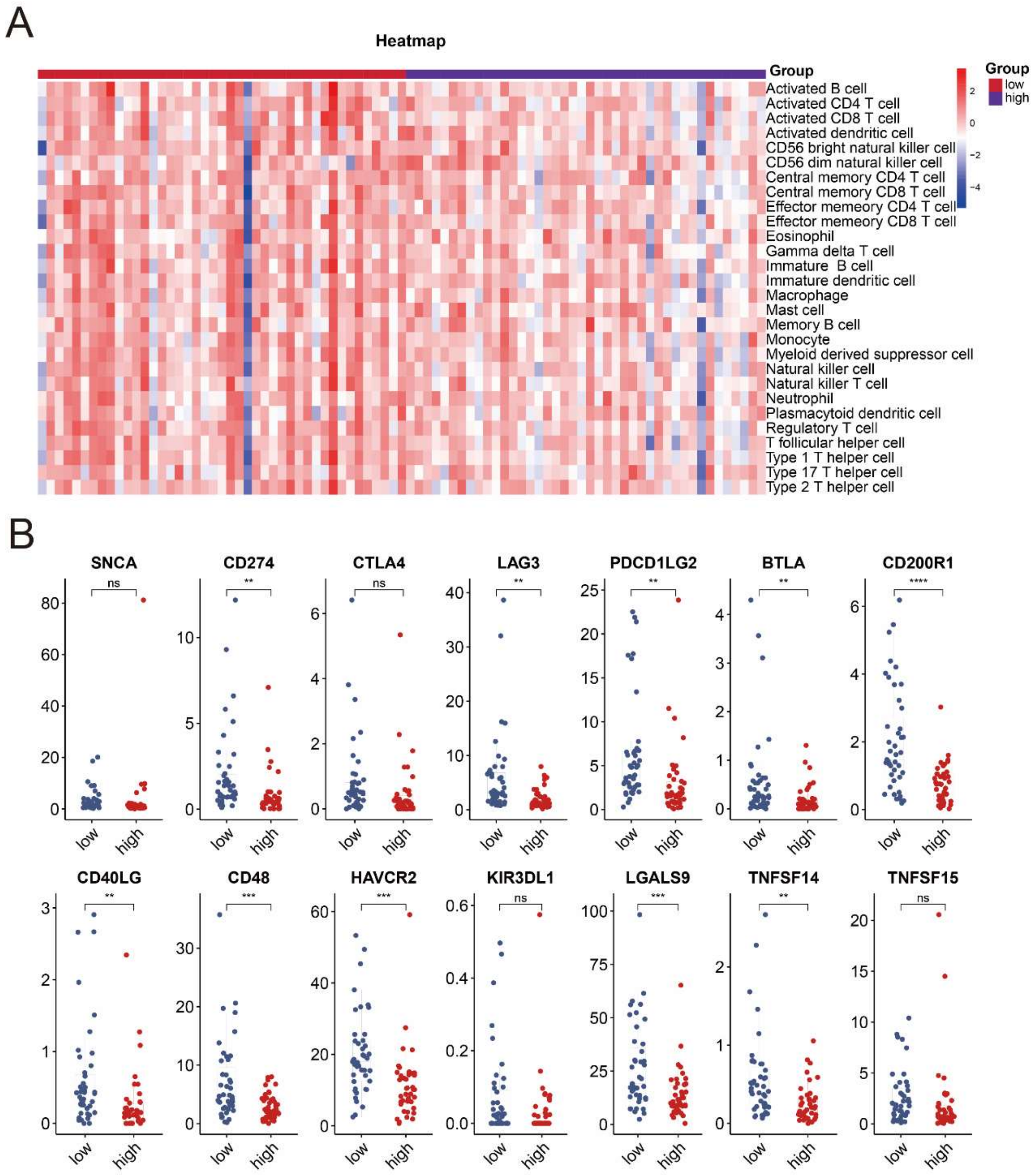

Sup Fig.3 Analysis of Immune Microenvironment and Immune Checkpoint. (A) heatmap of immune cells infiltration between high- and low-risk groups. **(B)** The expression levels of immune checkpoint-related genes.

Sup List 1, DRGs identified by WGCNA analysis

SIRPG	CD3D	FAIM3	TMEM176	MYO1F	TMEM176A	RASSF5	CD69	INPP5D	LGALS9
MS4A4E	TBC1D10C	ARHGEF15	CTSW	IRF1	VWA1	CD7	CPNE2	NRP1	GPR183
TNFRSF1B	SH3TC1	SH2D1A	CD37	FAM78A	CD34	SH3RF3	PECAM1	EVI2B	PRDM1
PLB1	KLRB1	CLEC14A	C19orf35	VNN2	CSF1	IL32	LY86	FRAT1	SPTBN5
ADCY4	PARVG	GYPC	CLDN5	MRO	LMO2	ACSL5	TLR7	TM4SF1	IFI6
DPEP2	SIRPB2	CXCL12	N4BP2L1	SIGLEC11	ASCL2	P2RY13	PILRA	CLEC7A	GNA14
BRICD5	LILRB5	CAPN11	GPR132	CD209	GIMAP5	VENTX	TRIM13	CCR5	BCAR3
MYC	TRPM2	TRAF3IP3	GIMAP8	PARP10	CCM2L	TLR6	GPR116	FAM105A	APLNR
CD2	LIMD2	GPSM3	MS4A14	GRAP	C3	TOR4A	GPR56	TIE1	AIF1
FOXP3	MMP15	GSDMD	HLA-DPA1	GPR65	TREML1	PCED1B	GAPT	HMHA1	SLAMF1
GIMAP1	MT1X	NUAK2	C16orf86	TANGO2	GLB1L2	LRMP	SLC15A3	SLCO2B1	CCDC88B
RASL10A	CMKLR1	STAB1	CIITA	CD74	MS4A4A	UNC13D	LAIR1	MANSC1	SIGLEC10
PIK3R6	SLA2	CFP	LGALS2	CBX7	MYO1G	LY6H	GZMK	CLEC4G	SOWAHD
IL24	USHBP1	VAV1	IL2RA	FAM124B	GRAP2	LILRB2	NOTCH4	CHST1	TBXAS1
DOK2	CD27	ADM5	GPIHBP1	CLEC10A	S1PR4	TMEM255B	NPR1	FLI1	IL17RA
ITGAL	LAG3	OLFML2A	TMEM150	FAM167B	MFNG	SP140	ANKRD22	SEMA4A	SLC43A2
NLRC3	LPAR6	EBI3	FMNL1	HLA-DRB1	IFI44	TBX21	CYP2S1	CECR6	PTAFR
ARRDC5	CD200R1	FES	PIK3CD	ROBO4	C10orf54	GPR18	VASH1	DENND1C	LILRB3
CSF3R	LYL1	TNFRSF14	SIGLEC14	HSH2D	HLA-DMB	COL5A3	SEPT1	CD14	NPL
RASAL3	LTB	CLEC3B	AOAH	ZNF683	PODNL1	NRROS	SLAMF8	RBP7	THEMIS2
CETP	PRAM1	HLA-DPB1	CFH	PDCD1	GIMAP6	GMFG	LRRC25	VMO1	CD52
SIT1	GIMAP7	RGS18	HBEGF	F13A1	TNFRSF10C	PDCD1LG2	C1orf162	MAP4K1	GPR141
SOX17	BIN2	CASP10	SPOCK2	CDH5	ABI3	PSMB9	CD300LF	P2RY12	GIMAP4
CD5	NLRP3	LILRB1	IKZF1	NHLRC4	BCL6B	BTN3A1	CD93	FLT3LG	IL1B
CXCR3	FGD2	NKG7	IL10	APOBEC3H	C1QB	PRSS36	LCP2	MPEG1	AMICA1
TMC8	PRND	IL10RA	MS4A6A	APOL3	VSIG4	PTPN7	C1QC	CXorf36	TNFSF13B
CXCR6	CORO7	GZMA	CD40	ARRB2	GJA4	FAM110A	HLA-DMA	PDE2A	PCDH12
PRF1	CD3E	PPP1R16B	JAG1	CSF2RA	EPHB1	HLA-DQA1	MYCT1	TMEM156	FCGR2A
POU2F2	METTL7A	MAPK15	HLA-DRA	CCR2	ZMYND15	ARHGAP27	SIGLEC1	SASH3	ITGAM
LCK	CARD11	CEBPA	KANK3	SECTM1	IL3RA	PPARGC1B	CD247	IER3	PTPN6
CD3G	KCNAB2	GAS6	CD79B	MRC1	FCGR1B	FCN1	TLR8	GPBAR1	AFAP1L2
MILR1	GZMH	TNS2	C1QA	MS4A7	IL12RB1	LEPR	SNX20	ESAM	RHOH
TLR4	S1PR1	APBB1IP	WAS	APOL1	CX3CL1	SLAMF6	PIK3R5	KIFC3	GAB3
C2	GPR82	NOVA2	SH2D3C	ZAP70	HLA-F	TAGAP	JADE2		

Sup List 2, prognostic DRGs by uni-COX analysis

Gene	coef	HR	HRl_5	HRh_95	p_value
CFH	-0.5245	0.591849	0.437228	0.801149	0.000686
WAS	-0.43491	0.647321	0.46375	0.903558	0.010589
BTN3A1	-0.68519	0.503993	0.308992	0.822055	0.006052
KCNAB2	-1.32111	0.266838	0.118316	0.601798	0.001453
VASH1	-0.62107	0.537369	0.348173	0.829373	0.005034
TBX21	-0.82108	0.439956	0.206344	0.938047	0.033543
APBB1IP	-0.42933	0.650942	0.483342	0.876658	0.004704
SP140	-1.16132	0.313071	0.121718	0.805251	0.015982
LAG3	-0.57459	0.562933	0.341879	0.926918	0.023932
CD209	-0.3854	0.680181	0.477908	0.968065	0.032339
MFNG	-0.49012	0.612554	0.375405	0.999513	0.049773
APOL1	-0.2783	0.757067	0.589718	0.971905	0.028996
LILRB5	-0.50016	0.606433	0.381814	0.963193	0.034105
CXCL12	-0.35237	0.70302	0.525979	0.939654	0.017291
MS4A4A	-0.37541	0.68701	0.505062	0.934504	0.01678
CD5	-0.87976	0.414881	0.206441	0.833778	0.013495
CSF3R	-0.50051	0.606219	0.369288	0.995162	0.0478
F13A1	-0.38287	0.681905	0.531501	0.87487	0.0026
FAM78A	-0.55574	0.573648	0.361199	0.911055	0.01854
LILRB2	-0.37974	0.684041	0.470817	0.99383	0.046323
IL2RA	-0.72758	0.483077	0.273246	0.85404	0.012326
IL10	-0.71956	0.486964	0.24022	0.987156	0.045953
GYPC	-0.38351	0.681464	0.483296	0.960887	0.028703
TLR4	-0.34279	0.709789	0.510408	0.987055	0.041609
MYC	0.727953	2.070837	1.405242	3.051691	0.000234
IFI44	-0.38811	0.678335	0.488038	0.942835	0.020868
GPR65	-0.53265	0.587049	0.357144	0.964951	0.03567
PIK3R5	-0.53806	0.58388	0.352388	0.967446	0.03676
VAV1	-0.42981	0.650632	0.433858	0.975715	0.037629
DOK2	-0.43286	0.648651	0.436699	0.963475	0.03201
GLB1L2	0.313906	1.368762	1.11516	1.680036	0.002677
VSIG4	-0.3126	0.731541	0.5734	0.933297	0.011888
GAB3	-0.65785	0.517964	0.296935	0.903521	0.020484
SLAMF6	-0.47823	0.619881	0.399184	0.962596	0.033193
PLB1	-0.9536	0.38535	0.156107	0.951237	0.038603
C2	-0.42452	0.654086	0.494511	0.865155	0.002929
SNX20	-0.74178	0.476266	0.268925	0.843464	0.010967
SLC43A2	-0.53933	0.583136	0.364016	0.934156	0.02488
ITGAM	-0.47216	0.623656	0.422763	0.920012	0.017301
CXCR6	-0.86467	0.42119	0.178014	0.99656	0.04909
SH3RF3	-0.47581	0.621383	0.409535	0.942819	0.025302
TNFRSF1	-0.76424	0.465687	0.265615	0.816462	0.007635
NRROS	-0.49773	0.607906	0.384468	0.961199	0.033233
CMKLR1	-0.418	0.65836	0.455516	0.951533	0.026126
PCED1B	-0.62659	0.534412	0.321192	0.889174	0.015859
GAS6	-0.35477	0.701336	0.492463	0.998799	0.049226
FMNL1	-0.45734	0.632968	0.406695	0.985131	0.04273
METTL7A	-0.50887	0.601177	0.39808	0.907893	0.015547
EVI2B	-0.38471	0.680647	0.51162	0.905515	0.008256
GJA4	-0.41757	0.658645	0.466606	0.929721	0.017582
ACSL5	-0.47814	0.619936	0.388288	0.989782	0.045178
CD3E	-0.3742	0.687842	0.475351	0.995321	0.047162
LILRB3	-0.69539	0.498878	0.273213	0.910934	0.023597
GPSM3	-0.41258	0.661938	0.456123	0.960621	0.029901
LTB	-0.40715	0.665541	0.444654	0.996156	0.047852
CEBPA	-0.49762	0.607975	0.425318	0.869074	0.006339
